# Abietic acid targets RAN to restrict coxsackievirus B3 infection

**DOI:** 10.3389/fmicb.2026.1874898

**Published:** 2026-06-16

**Authors:** Junbo Huang, Qing Song, Yanjun Di, Zhiyun Cheng, Zhaocheng Yang, Xibo Lin, Kaiyuan Hunag, Haoyi Zhan, Lijuan Xie, Jieqing Liu, Lei Tong

**Affiliations:** School of Medicine, Huaqiao University, Quanzhou, China

**Keywords:** abietic acid, antiviral, coxsackievirus B3, phosphorylated STAT1/2, Ras-related nuclear protein

## Abstract

Coxsackievirus B3 (CVB3) represents a major etiological factor of viral myocarditis (VMC). It frequently results in the damage of the heart and even heart failure, but there are few effective treatment measures that can be used clinically. In this paper, we show that abietic acid (AA), a tricyclic diterpenoid compound, has strong anti-CVB3 activity both *in vitro* and *in vivo*. AA was found to inhibit CVB3 replication with 50% effective concentration (EC50) of 12.16 μM and high selectivity index (SI = 26.90) and 50 percent cytotoxic concentration (CC50) of 327.14 μM in HeLa cells, suggesting good biosafety. Mechanistically, RAN (Ras-related nuclear protein) a central mediator of nucleocytoplasmic transport was determined to be an essential host dependency factor in CVB3 replication. The AA therapy led to a significant decrease in RAN expression, thus recovering nuclear translocation of phosphorylated STAT1/2 (p-STAT1/2) compromised by CVB3 infection, which subsequently boosts the response of interferon-stimulated genes (ISGs) downstream. AA treatment in a CVB3-induced model of murine myocarditis showed a significant increase in survival rates, lower viral loads and inflammatory cytokines in cardiac tissues, and lessened myocardial injury. Collectively, these results identify RAN as a novel antiviral target, and show that AA inhibits CVB3 infection via the RAN-p-STAT1/2 axis, supporting its potential as a promising therapeutic agent for CVB3-associated diseases.

## Introduction

Coxsackievirus B3 (CVB3) is a member of the family Picornaviridae and the genus *Enterovirus*. It is also one of the key pathogens that cause viral myocarditis (VMC; Garmaroudi et al., 2015). Epidemiological research has demonstrated that CVB3 infection causes 25%–40% of cases of acute myocarditis, especially in the young population (Daba et al., 2019). CVB3 infection leads to the destruction of myocardial cells, local inflammatory invasion, and ventricular remodeling. It may even progress to dilated cardiomyopathy and heart failure in severe cases, placing a significant burden on healthcare systems (Nappi, 2025). Nevertheless, there are no particular antiviral medications available at present to treat CVB3 infection. Clinical intervention primarily involves supportive treatment and immunomodulation, which have limited effectiveness and do not inhibit the replication of viruses fundamentally (Tian et al., 2024). Thus, developing effective and low-toxicity anti-CVB3 agents is of great clinical significance.

Natural products, because of their structural variety and wide-ranging biological activities, have long been important sources for antiviral drug discovery (Salampe et al., 2023). Abietic acid (AA) is a tricyclic diterpene compound that is widely present in the resins of coniferous plants and represents the main active component of rosin (Ren et al., 2025). Growing evidence has confirmed the multiple pharmacological properties of AA, such as its anti-inflammatory, antibacterial and anti-tumor activities (Ahmad et al., 2024, An et al., 2023, Faustino et al., 2018). Currently, its antiviral potential has received increasing attention. Studies show that AA derivatives display inhibitory effects against influenza A (IAV) and severe acute respiratory syndrome coronavirus 2 (SARS-CoV-2; Tret’yakova et al., 2022; Tretyakova et al., 2023). However, whether AA shows anti-CVB3 activity and its underlying mechanism remain elusive, with no related studies reported so far.

As a small GTPase from the Ras superfamily, RAN (Ras-related nuclear protein) has essential functions in nuclear-cytoplasmic transport, cell cycle regulation, spindle assembly and signal transduction (El-Tanani et al., 2023; Moore, 2001). Moreover, RAN facilitates the transmembrane transfer of proteins with nuclear localization signals or nuclear export signals by interacting with its effector molecules (Güttler and Görlich, 2011). It is increasingly being shown that RAN participates in the replication of different viruses. For example, HIV-1 Rev protein uses RAN-mediated nuclear export to promote viral mRNA transport (Smith et al., 2025; Taltynov et al., 2013), whereas the nuclear export of viral ribonucleoprotein complexes is controlled by influenza virus NS2 protein via interactions with RAN (Cao et al., 2024). Accumulating studies have further clarified the pivotal involvement of RAN in the replication of picornaviruses and enteroviruses. Mechanistically, host microRNAs could target RAN to exert regulatory effects on *Enterovirus* infection. Tang et al. (2016) reported that miR-197 negatively modulates the infectious cycle of *Enterovirus* 71 by directly targeting RAN protein. Consistently, Orr-Burks et al. (2017) verified that miR-134 serves as an important regulator affecting poliovirus and *Enterovirus* 71 infection through RAN-related pathways. However, the functional role of RAN in CVB3 infection has not been systematically characterized.

Activator of transcription 1/2 (STAT1/2), as transcription factors, are the central components in the type I interferon (IFN) signaling pathway and occupy a central position in host antiviral immunity (Tong et al., 2024; Yang et al., 2022). When viruses invade, type I IFNs attach to cell surface receptors, stimulate JAKs and then cause STAT1 and STAT2 phosphorylation. The phosphorylated form of STAT1/2 (p-STAT1/2) form heterodimers and binds with IRF9 to establish ISGF3 complex, which is then transported to the nucleus to induce interferon-stimulated genes (ISGs) transcription, thus creating an antiviral state (Zemke and Berk, 2017). One of the most common immune evasion strategies employed by viruses is interference with the nuclear translocation of STAT proteins. Indicatively, V protein of paramyxoviruses directly interacts with STAT proteins, while inhibiting their phosphorylation (Horvath, 2004; Ulane and Horvath, 2002), and NS5 protein of dengue virus induces degradation of STAT2 (Best, 2017). It is not well known whether CVB3 causes disruption of the STAT signaling pathway in a similar manner. Therapeutically promising drugs that inhibit the process of STAT nuclear translocation can be used to re-establish the host antiviral immunity and should be investigated.

In view of the above background, this study will examine the inhibitory effect of AA on CVB3 replication and its molecular mechanism, emphasizing the explanation of the functional role of RAN in viral replication and the regulatory role of AA in p-STAT1/2 nuclear translocation. In addition, we will assess its therapeutic efficacy against viral myocarditis using *in vivo* experiments, thereby offering new candidate molecules and a theoretical framework on which anti-CVB3 therapeutics can be developed.

## Materials and methods

### Reagents, viruses, and cells

Abietic acid (GC40831, purity > 98%) was purchased at GlpBio (Shanghai, China). Novoprotein (Shanghai, China) provided IFN-α (C005). HeLa cells (T8969) were obtained from Abmgood (Shanghai, China) and were cultured in the medium of HeLa cells (CL-0101, Procell, Wuhan). The CVB3 Woodruff strain (GenBank: U57056.1) and the recombinant virus EGFP-CVB3 (Tong et al., 2011) were kindly provided by Professor Zhaohua Zhong, Department of Microbiology, Harbin Medical University. Viral stocks were propagated in HeLa cells and stored at −80 °C.

### Cell viability assay

HeLa cells at the logarithmic growth phase were cultured in 96-well plates. Cells were incubated with increasing concentrations of AA after cell attachment for 48 h. At the end of treatment, 10 μL CCK-8 solution (CA1210, Solarbio, Beijing) was added to each well and incubated for 1 h at 37 °C. A Microplate reader was used to measure absorbance at 450 nm. Cell viability was determined as the percentage of absorbance when compared to the untreated controls.

### Virus titration

HeLa cells were digested and seeded in 96-well plates at the density of 2 × 10^3^ cells/well. The culture medium was discarded after 16 h of culture when cell monolayer was about to be 80 percent confluent and cells were washed three times with PBS. Virus stock was diluted in serum-free DMEM (C0891, Bytotime) in serial dilutions of 10-fold. Each virus dilution was inoculated into the cells with a volume of 100 μL and control wells were inoculated with maintenance medium only. After incubating the inoculum 2 h at 37 °C, the inoculum was removed and replaced with 100 μL of maintenance medium with 2% FBS. Plates were placed in an incubator at 37 °C and left to incubate for 72 h. Cytopathic effect (CPE) was determined microscopically and viral titers were estimated by Reed-Muench method.

### Co-immunoprecipitation and Western blot

HeLa cells transfected were lysed in ice-cold RIPA buffer (P0013, Beyotime, Shanghai) with the addition of protease inhibitor cocktail. Lysates were sonicated for 2 min and centrifuged at 12000 × *g* for 10 min at 4 °C. Supernatants were incubated with anti-HA tag antibody (AF0039, Beyotime) at 4 °C over 2 h, then pre-equilibrated Protein A magnetic beads (P2175S, Beyotime) were added to the supernatant and incubated further at 4 °C over 3 h. Beads were washed three times with pre-chilled RIPA buffer, and bound proteins were eluted by boiling in 2 × SDS loading buffer (P0015, Beyotime) for 10 min. Western blot analysis was done on the eluted samples.

For Western blot, total proteins were isolated in cells or tissues and measured by BCA method. Equal amounts of protein were separated by SDS-PAGE and transferred to PVDF membranes (ISEQ00010, Solarbio, Beijing, China). Membranes were incubated overnight at 4 °C after being blocked with 5% skim milk (P0216, Beyotime) at room temperature. The membranes were washed three times using TBST (ST673, Beyotime) and incubated with HRP-conjugated secondary antibodies at room temperature. Immunoreactive bands were seen after washing with a chemiluminescence detection system. The main primary antibodies were as follows: The primary antibodies used were as follows: GAPDH (10494-1-AP, Proteintech, Wuhan), Histone H3 (17168-1-AP, Proteintech), STAT1 (10144-2-AP, Proteintech), STAT2 (16674-1-AP, Proteintech), p-STAT1 (28977-1-AP, Proteintech), RAN (AG3073, Beyotime), HA (AF0039, Beyotime), p-STAT2 (AF5938, Beyotime), and CVB3 3D antibody (kindly provided by Professor Zhaohua Zhong). Secondary antibodies were HRP-conjugated goat anti-mouse (RGAM001, Proteintech) and HRP-conjugated goat anti-rabbit (RGAR001, Proteintech).

### RT-qPCR

Cells or cardiac tissues were lysed in TRIzol reagent (RN001, ESScience, Shanghai) to obtain total RNA. Total RNA was reverse-transcribed into cDNA with HiScript QRT SuperMix (RA103, Vazyme, Nanjing) using one microgram of total RNA. Taq Pro Universal SYBR qPCR Master Mix (Q712, Vazyme) was used for quantitative real-time PCR on a LightCycler 96 system. The thermal cycling parameters were as follows: initial incubation at 95 °C for 30 s, then 40 cycles of 95 °C incubation for 10 s and 60 °C incubation for 30 s. GAPDH served as an internal control. The 2^– ΔΔCt^ method was used to determine the relative levels of gene expression. Supplementary Table 1 contains primer sequences.

### Time-of-drug-addition assay

HeLa cells were cultured in 6-well plates at a concentration of 1 × 10^6^ cells/well. At approximately 80% confluence, cells were rinsed twice with PBS and subjected to three treatment regimens. For the pre-infection treatment, cells were pretreated with AA for 1 h, washed with PBS, and infected with CVB3 (MOI = 1) at 4 °C for 1 h; the virus inoculum was then removed, and cells were cultured in maintenance medium for 24 h. For the co-infection treatment, cells were infected with CVB3 (MOI = 1) in the presence of the indicated concentrations of AA at 4 °C for 1 h; the virus inoculum was then removed, and cells were washed with PBS and cultured in maintenance medium for 24 h. For the post-infection treatment, cells were infected with CVB3 (MOI = 1) at 4 °C for 1 h; the virus inoculum was then removed, and cells were treated with the indicated concentrations of AA for 24 h. Cell lysates were then collected to quantify viral genome copies using RT-qPCR.

### Knockdown and overexpression

Human RAN (Gene ID: 5901, NM_006325.5) was synthesized from Sangon (Shanghai). The siRNA sequences were: sense strand 5′-GUGGCAACAAAGUGGAUAUTT-3′ and antisense strand 5′-AUAUCCACUUUGUUGCCACTT-3′. HeLa cells were cultured in 6-well plates to approximately 50% confluence. Transfection of siRAN (50 nM) or siNC was performed using Lipofectamine 2000 (L7800, Solarbio, Beijing) according to the manufacturer’s instructions. Cells were harvested after 48 h for subsequent viral infection experiments.

To overexpress RAN, the entire coding sequence (651 bp) of human RAN was inserted into the pLenti-GIII-CMV-C-term-3xHA-CBH-GFP-2A-Puro lentiviral expression vector (Abmart, Shanghai). The construct was confirmed by sequencing. HeLa cells were cultured in 6-well plates to approximately 70% confluence. The pLenti-RAN overexpression plasmid or empty vector control was transfected using Lipofectamine 2000 according to the manufacturer’s instructions. At 48 h post-transfection, cells were used for subsequent viral infection assays.

### Molecular docking and molecular dynamics simulation

The human RAN protein (PDB ID: 1IBR) crystal structure was obtained in the RCSB Protein Data Bank, and the 3D AA structure was downloaded to PubChem as an SDF file and transformed into MOL2 format with the help of ChemDraw software. AutoDock Vina (version 1.5.7) was used to carry out molecular docking. Water molecules were eliminated, and polar hydrogens introduced to prepare the protein structure. The whole protein was used as a docking grid. Conformational sampling was done using genetic algorithm where 20 independent runs were carried out. The lowest binding energy score was used to choose the best docking pose. Docking values were analyzed and visualized by PyMOL (version 3.0.3) and Schrodinger Maestro (version 14.0).

The optimal protein-ligand complex was simulated with molecular dynamics (MD) using GROMACS (version 2024.2), Amber99sb (Smith et al., 2015) force field, and TIP3P water model. The Na^+^ or Cl^–^ ions were used to neutralize the system. Steepest descent algorithm was used in energy minimization. The system was subsequently equilibrated at NVT and NPT ensembles (300 K, 1 bar) over a period of 100 ps each. Lastly, the binding stability of the complex was determined by running a 100 ns MD simulation.

### Animal experiments

Five-week-old male BALB/c mice were purchased from Wu Experimental Animal (Fujian, China). All animal experimental protocols were approved by the Experimental Animal Ethics Committee of Huaqiao University (approval number: A2025150). The three groups (*n* = 9) were randomly divided into normal control (Mock), virus control (CVB3), and AA treatment (CVB3/AA). To establish a viral myocarditis model, mice in the virus control and AA treatment groups were intraperitoneally injected with CVB3 (1 × 106 TCID50), whereas the normal control group received an equal volume of PBS. At 12 h post-infection, mice in the treatment group were administered AA (100 mg/kg/day) intraperitoneally for 7 days. Control mice received the same volume of vehicle. Daily monitoring of body weight and survival was performed.

Blood samples were taken on day 7 after infection. The activity of CK-MB and LDH was determined using serum as directed by the manufacturer. Heart tissues were harvested and cut into two different parts: one was fixed in 4% paraformaldehyde to be used in histopathological analysis, whereas the other was snap-frozen in liquid nitrogen to extract RNA and proteins. HE-stained paraffin sections of heart were viewed under a light microscope to detect pathological changes.

### Statistical analysis

Data are shown as mean ± SD. Statistical analyses were performed using GraphPad Prism 8. Unpaired Student’s *t*-test and one-way ANOVA with Tukey’s *post hoc* test were used for two-group and multiple-group comparisons, respectively. Differences were considered significant at *P* < 0.05.

## Results

### AA suppresses CVB3 replication *in vitro*

**FIGURE 1:**
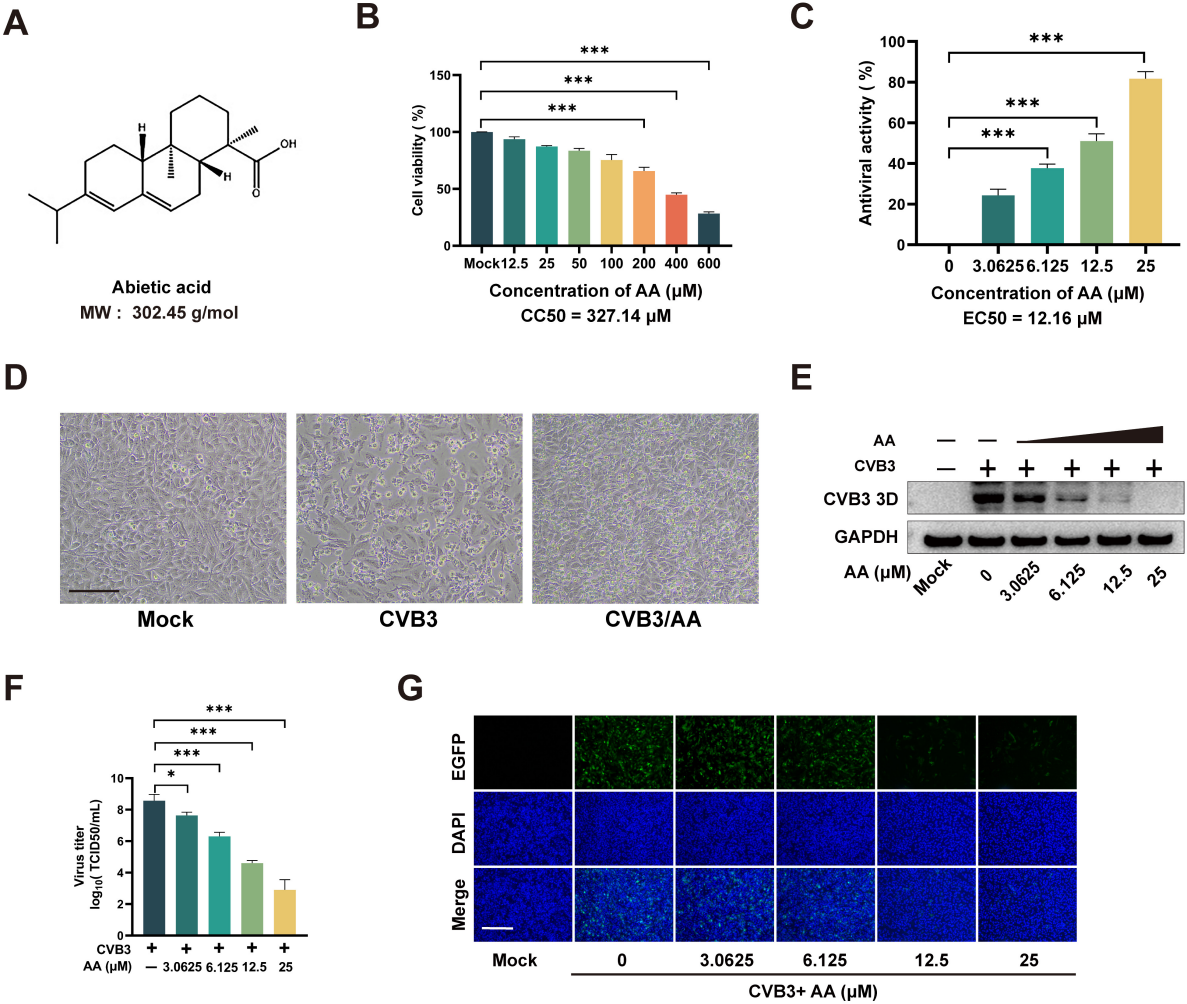
Abietic acid (AA) exhibits potent antiviral activity against CVB3 *in vitro*. **(A)** The chemical structure of AA. **(B)** The indicated concentrations of AA were employed for the treatment of HeLa cells over a period of 48 h, and CCK-8 assay was performed to measure cell viability. **(C)** HeLa cells were infected with CVB3 (MOI = 1) and then treated with increasing concentrations of AA for 24 h. RT-qPCR was utilized to determine viral genome copy numbers. **(D)** HeLa cells underwent infection using CVB3 (MOI = 1) and treated via 25 μM AA over a period of 24 h. Morphological variations were detected using a light microscope. Scale bar = 10 μm. **(E)** HeLa cells were inoculated with CVB3 at an MOI of 1, followed by treatment with indicated concentrations of AA over a period of 24 h. The protein expression level of CVB3 3D was analyzed via Western blot analysis, with GAPDH used as the loading control. **(F)** Cells and culture supernatants from E were gathered, and viral titers were identified using TCID50 assay. **(G)** HeLa cells were inoculated with EGFP-CVB3 at an MOI of 5, followed by treatment with the indicated concentrations of AA over a period of 24 h. EGFP fluorescence was visualized utilizing a fluorescence microscope. Scale bar = 10 μm. Values are presented as mean ± SD. *P < 0.05, ***P < 0.001.

The tricyclic diterpene compound (C_20_H_30_O_2_) AA has the chemical structure shown in Figure 1A. CCK-8 assay was hereby adopted to determine the cytotoxicity of AA on HeLa cells initially. There was no significant cytotoxicity at concentrations lower than 12.5 μM and CC50 was found to be 327.14 μM (Figure 1B), indicating a good safety profile within the concentration range tested. The antiviral effect of AA against CVB3 was then measured. AA suppressed CVB3 replication in a dose-dependent manner, with an inhibition rate of 81.7% at 25 μM and an EC50 of 12.16 μM (Figure 1C). The selectivity index (SI = CC50/EC50) was determined to be 26.90, indicating low toxicity and broad therapeutic window.

The protective effect of AA was also confirmed by microscopic observation of cell morphology. Normal HeLa cells exhibited distinct boundaries and minimal debris in the culture supernatant. Normal HeLa cells exhibited distinct boundaries and minimal debris in the culture supernatant. These cytopathic effects were markedly attenuated by AA treatment, restoring normal cell morphology (Figure 1D).

To validate the antiviral effect at molecular levels, Western blotting was employed to investigate the expression of CVB3 3D. An intense CVB3 3D signal was observed in cells infected with CVB3, which showed active viral replication. AA treatment induced a dose-dependent decrease in CVB3 3D levels and almost complete inhibition at 25 μM (Figure 1E). As expected, the viral titers were reduced in a dose-dependent manner upon AA treatment, and a reduction of 66% at 25 μM was achieved compared to the infected control (Figure 1F). Moreover, cells infected with EGFP-CVB3 showed high-intensity green fluorescence, indicating strong viral growth. The intensity of fluorescence decreased in a dose-dependent fashion following AA treatment, and no significant signal could be detected at 25 μM (Figure 1G). Taken together, these findings indicate that AA has a marked inhibitory effect on the *in vitro* replication of CVB3.

### AA inhibits CVB3 entry and post-entry replication stages

**FIGURE 2:**
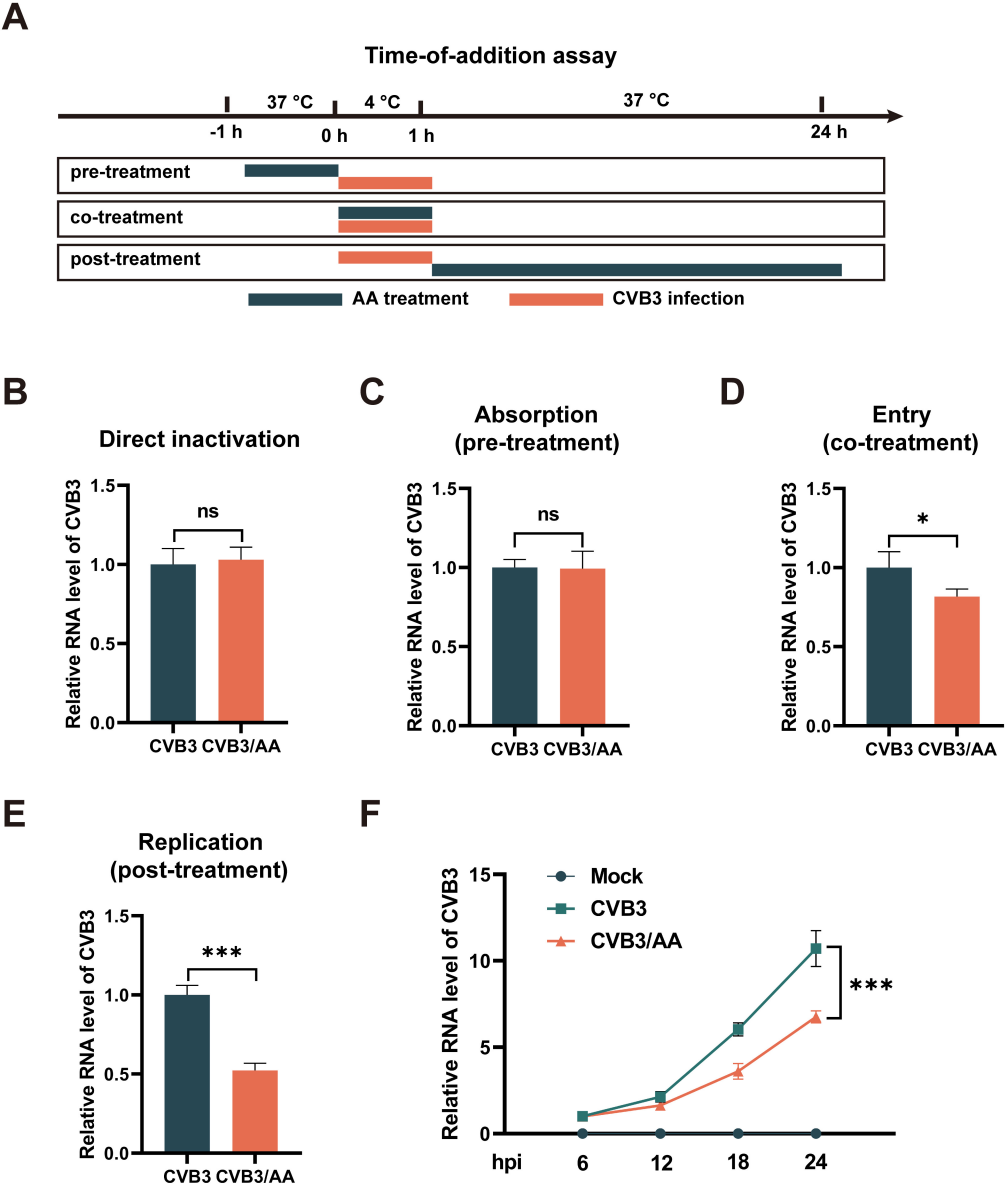
AA blocks CVB3 entry and replication. **(A)** Schematic illustration of the time-of-drug-addition assay. **(B)** CVB3 was pre-incubated using 25 μM AA over a period of 1 h ahead of infection of HeLa cells. Viral genome copy numbers were assessed using RT-qPCR. **(C)** HeLa cells underwent pretreatment using 25 μM AA for over a period of 1 h, followed by washing, and then infection with CVB3 (MOI = 1). Viral genome copy numbers were determined by RT-qPCR. **(D)** HeLa cells underwent infection with CVB3 (MOI = 1) in the presence of 25 μM AA at 4 °C for 1 h. Unbound virus was removed by washing, and viral genome copy numbers were determined by RT-qPCR. **(E)** HeLa cells underwent infection with CVB3 (MOI = 1) for 1 h, then treated with 25 μM AA for 24 h. Viral genome copy numbers were calculated using RT-qPCR. **(F)** Viral growth curve. Values are presented as mean ± SD. ns, not significant; *P < 0.05, ***P < 0.001.

Time-of-addition assays were conducted to define the phases of the CVB3 life cycle that are affected by AA. HeLa cells were treated with three treatment regimens: pre-infection (AA before viral adsorption), co-treatment (AA during adsorption) and post-infection (AA after adsorption) as shown in Figure 2A. AA did not show any direct virucidal activity against CVB3 (Figure 2B) and had no effect on virus adsorption (Figure 2C). Nevertheless, AA strongly suppressed viral entry when added during adsorption (Figure 2D) and inhibited viral replication when added during the intracellular phase (Figure 2E). Analysis of viral growth curves showed that AA treatment markedly reduced viral genome copy number at 24 h post-infection compared with the virus control group (Figure 2F). Such findings reveal the antiviral action of AA through interference with both viral entry and later intracellular replication stages.

### AA suppresses CVB3 infection by downregulating RAN

**FIGURE 3:**
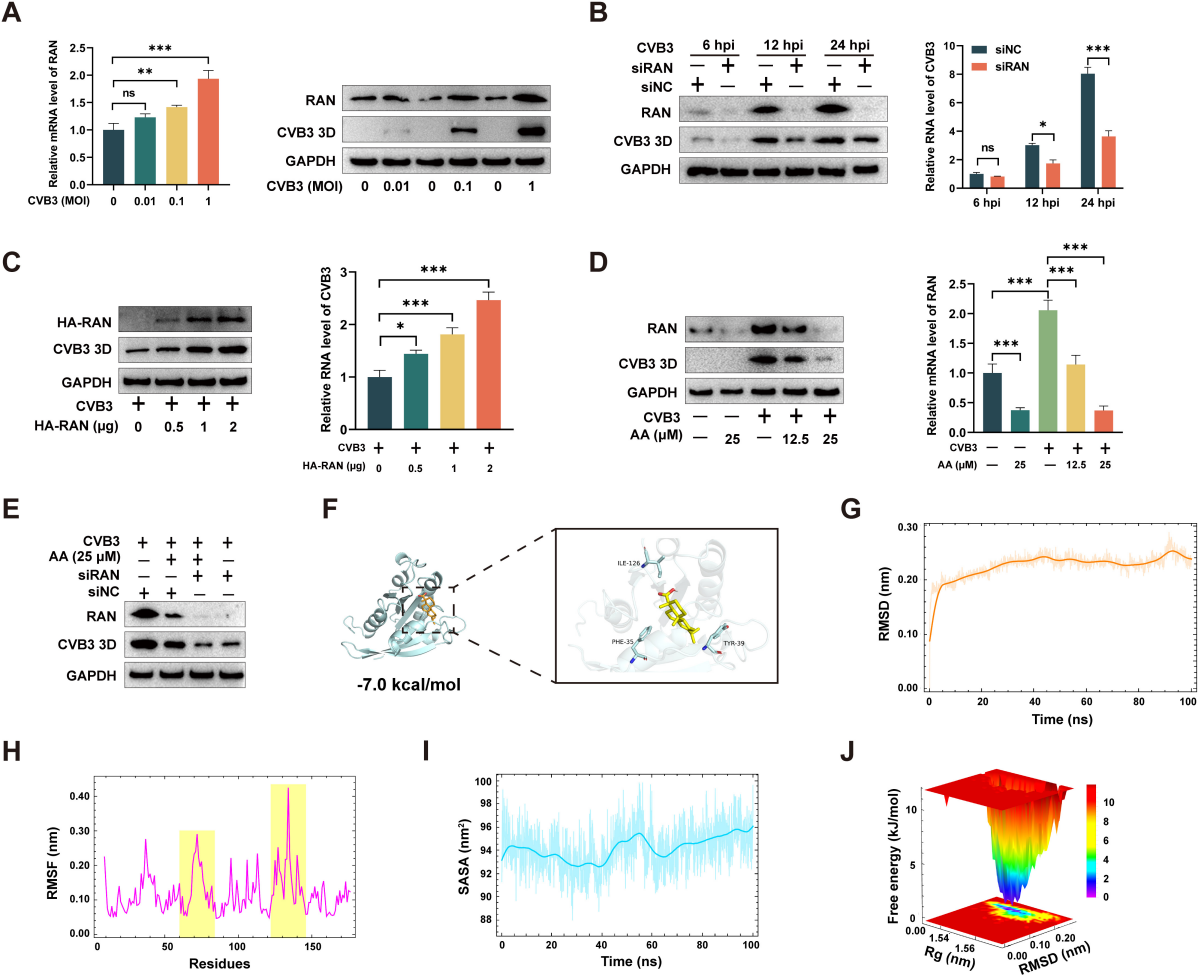
AA directly targets RAN and downregulates its expression to inhibit CVB3 replication. **(A)** HeLa cells experienced infection with CVB3 at indicated MOIs over a period of 24 h. RAN mRNA levels were measured by RT-qPCR, and protein levels of RAN and viral 3D were identified via Western blot. GAPDH served as a loading control. **(B)** HeLa cells underwent transfection with siNC or siRAN for 48 h, then infected with CVB3 (MOI = 1) for 24 h. Viral genome copy numbers were measured using RT-qPCR, and protein levels of RAN and viral 3D were identified via Western blot. **(C)** HeLa cells underwent transfection with pLenti-vector or increasing doses (0.5, 1, 2 μg) of pLenti-RAN over a period of 48 h, and then were infected infected with CVB3 (MOI = 1) for 24 h. Viral genome copy numbers were measured by RT-qPCR, and protein levels of HA, RAN, and viral 3D were detected by Western blotting. **(D)** HeLa cells underwent infection with CVB3 (MOI = 1) over a period of 2 h, followed by treatment with 25 μM AA for 24 h. RAN mRNA levels were measured by RT-qPCR, and protein levels of RAN and viral 3D were evaluated via Western blotting. **(E)** RAN knockdown abrogates AA’s antiviral effect. HeLa cells underwent transfection with si-NC or si-RAN, followed by infection with CVB3 (MOI = 1) and treatment with 25 μM AA. RAN and viral 3D protein levels were detected via Western blot. **(F–J)** Molecular docking and dynamics simulations of AA binding to RAN. Data are expressed as mean ± SD. *P < 0.05, ***P < 0.001.

To examine the possibility that CVB3 infection regulates host factor RAN expression, HeLa cells underwent infection with CVB3 at various multiplicities of infection (MOI = 0.01, 0.1, 1). RAN mRNA and protein levels rose in a dose-dependent fashion after CVB3 infection (Figure 3A), indicating that CVB3 infection causes RAN upregulation.

The loss- and gain-of-function studies were used for analyzing the functional role of RAN in CVB3 replication. Specific siRNA knockdown of RAN significantly decreased the copy number of CVB3 genomic RNA and the expression of CVB3 3D (Figure 3B). In contrast, transfection of increasing doses (0.5, 1, 2 μg) of the RAN plasmid (encoding HA-tagged RAN) led to a dose-dependent increase in viral RNA and CVB3 3D levels (Figure 3C). These findings identify RAN as a host dependency factor required for efficient CVB3 replication.

Considering the antiviral activity of AA and the proviral effect of RAN, we then investigated whether AA acts by regulating RAN expression. AA treatment of CVB3-infected HeLa cells significantly decreased both RAN mRNA and protein levels and was associated with a reduction in viral 3D (Figure 3D). Interestingly, AA treatment also decreased RAN expression in uninfected cells, indicating that AA has a direct effect on RAN. To investigate whether RAN downregulation is important for the antiviral activity of AA, rescue experiments were carried out in RAN-knockdown cells. Knockdown of RAN alone suppressed CVB3 3D expression, and AA treatment did not further reduce CVB3 3D levels in RAN-deficient cells (Figure 3E), suggesting that the antiviral activity of AA is primarily mediated by RAN.

Molecular docking studies showed that AA binds to RAN with a binding energy of −7.0 kcal/mol and forms hydrophobic interactions with residues Phe35, Tyr39 and Ile126 (Figure 3F). The stability of the AA-RAN complex was determined by 100 ns molecular dynamics simulations. The root-mean-square deviation (RMSD) analysis revealed that the complex stabilized with fluctuations around 0.24 nm (Figure 3G). The root-mean-square fluctuation (RMSF) analysis uncovered higher flexibility in residues 60–85 and 120–150 which may be involved in ligand adaptation (Figure 3H). The solvent-accessible surface area (SASA) analysis did not show any significant conformational changes in the hydrophobic core (Figure 3I). The free energy landscape showed only one minimum energy cluster, indicating convergence to stable conformation (Figure 3J). These results indicate that AA directly interacts with RAN, downregulates its expression and thus inhibits CVB3 replication.

### AA restores STAT1/2 nuclear import in a rAN-dependent manner

**FIGURE 4:**
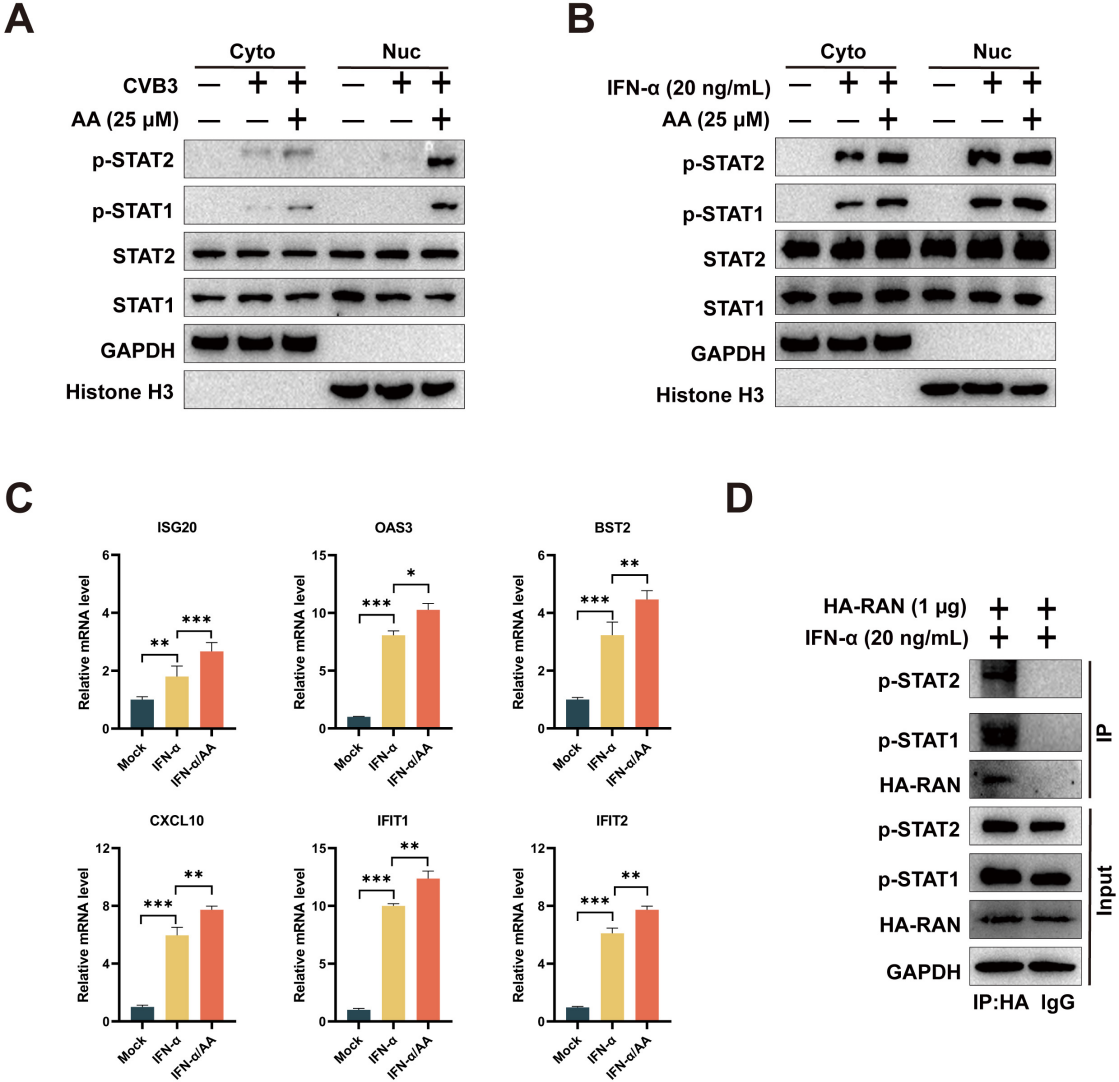
AA restores STAT1/2 nuclear import in a RAN-dependent manner. **(A)** HeLa cells underwent infection with CVB3 (MOI = 1) and treatment with 25 μM AA for 24 h. Nuclear and cytoplasmic fractions were isolated, and the distribution of p-STAT2, p-STAT1, STAT1, and STAT2 was analyzed by Western blotting. Histone H3 and GAPDH served as nuclear and cytoplasmic markers, respectively. **(B)** HeLa cells experienced treatment with 25 μM AA, IFN-α, or their combination. Nuclear fractions were isolated, and p-STAT1/2 levels were detected by Western blot. **(C)** HeLa cells experienced treatment with 25 μM AA, IFN-α, or their combination over a period of 24 h. ISGs mRNA levels were measured via RT-qPCR. **(D)** Lysates from HeLa cells overexpressing HA-RAN were immunoprecipitated with anti-HA antibody. The immunocomplexes were analyzed by Western blot using antibodies against p-STAT2, p-STAT1, and HA. Input and IgG controls are presented. Values are presented as mean ± SD. *P < 0.05, **P < 0.01, ***P < 0.001.

In accordance with the well-documented role of RAN in nucleocytoplasmic transport, we proposed that AA could affect the subcellular distribution of p-STAT1/2. Immunoblotting after nuclear and cytoplasmic fractionation showed that CVB3 infection considerably decreased nuclear p-STAT1/2 levels but enhanced their cytoplasmic accumulation, which indicated that the virus interfered with STAT nuclear translocation. Nuclear p-STAT1/2 levels were restored by AA treatment in cells infected with CVB3 (Figure 4A), indicating that AA rescues the defect caused by the virus in STAT nuclear import.

To investigate possible synergy with type I interferon signaling, HeLa cells underwent treatment with AA, IFN-α or both. The combined treatment also increased nuclear accumulation of p-STAT1/2 over each agent alone (Figure 4B). As expected, AA synergized with IFN-α to induce higher mRNA expression of interferon-stimulated genes than that induced by IFN-α alone (Figure 4C).

To explain the molecular interaction that leads to AA-mediated STAT nuclear retention, co-immunoprecipitation assays were carried out. Immunoprecipitation with anti-HA antibody in lysates of HeLa cells overexpressing HA-RAN co-precipitated endogenous p-STAT1 and p-STAT2, indicating a physical interaction between RAN and phosphorylated STATs (Figure 4D). These findings indicate that AA facilitates p-STAT1/2 nuclear retention by preserving the function of RAN, which enhances antiviral signaling and inhibits CVB3 replication.

### AA protects against CVB3-induced VMC *in vivo*

**FIGURE 5:**
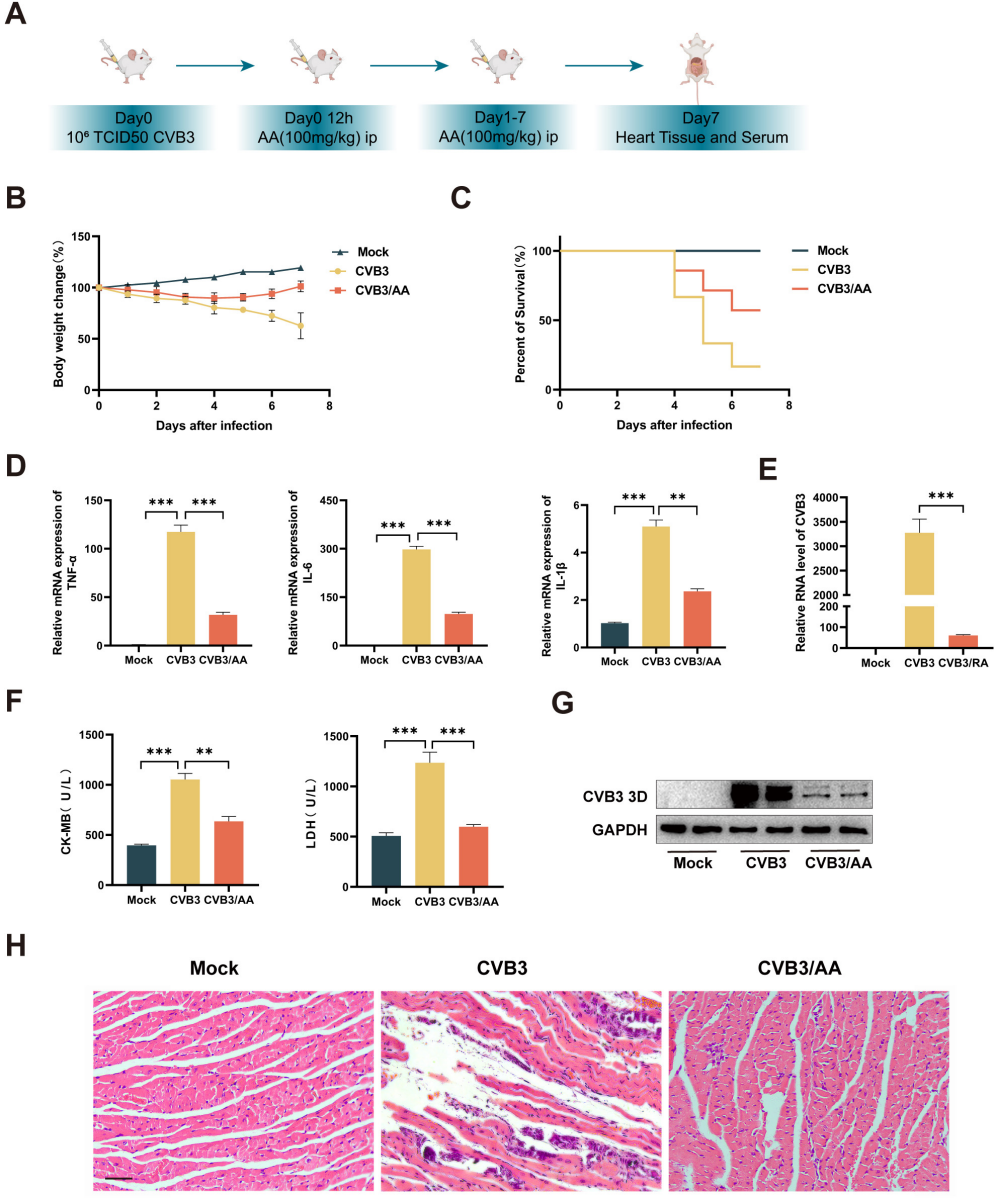
*In vivo* antiviral efficacy of AA against CVB3-induced myocarditis. **(A)** Schematic illustration of the *in vivo* experimental design. **(B)** Mice were weighed daily, and body weights were normalized to initial values. **(C)** Survival curves of mice in each group over 7 days post-infection. **(D)** Heart tissues were collected at 7 dpi, and mRNA levels of IL-6, TNF-α, and IL-1β were measured by RT-qPCR. **(E)** Heart tissues were collected at 7 dpi, and CVB3 genome copy numbers were detected via RT-qPCR. **(F)** Serum was collected at 7 dpi, and CK-MB and LDH activities were measured. **(G)** Heart tissue lysates were prepared at 7 dpi, and viral 3D protein expression was analyzed using Western blotting. **(H)** Heart sections were stained utilizing HE at 7 dpi and examined under a light microscope. Scale bar = 10 μm. Values are expressed as mean ± SD. *P < 0.01, ***P < 0.001.

In a CVB3-induced myocarditis mouse model, AA was evaluated for its antiviral activity *in vivo*. BALB/c mice (5 weeks old) were infected with CVB3 and were administered AA (100 mg/kg) or vehicle at 12 h post-infection, once daily for 7 days (Figure 5A). The 7-day survival rate was significantly higher in the AA-treated group than in the virus control group (Figure 5B). In addition, AA-treated mice lost less body weight and recovered more quickly (Figure 5C).

Abietic acid treatment significantly reduced CVB3 genomic RNA copy numbers in myocardial tissues (Figure 5E) and inhibited the expression of CVB3 3D (Figure 5G), indicating effective inhibition of viral replication in the target organ. Moreover, AA treatment suppressed pro-inflammatory cytokines (TNF-α, IL-6, IL-1β) upregulation in the heart caused by CVB3 (Figure 5D) and reduced serum concentrations of cardiac injury markers CK-MB and LDH (Figure 5F), suggesting amelioration of myocardial damage. HE staining histopathological examination showed that AA treatment significantly minimized the damage to the myocardium caused by CVB3 such as myofiber degeneration, necrosis, and inflammatory cell invasion (Figure 5H). These findings indicate that AA administration effectively inhibits CVB3 replication *in vivo*, reduces virus-induced inflammatory responses, and prevents myocardial injury.

## Discussion

Coxsackievirus B3 is a major etiological factor of VMC, a disease characterized by destruction of myocardial cells, local inflammatory infiltration, and ventricular remodeling in predisposed individuals. In severe cases, CVB3 infection may also give rise to dilated cardiomyopathy as well as heart failure (Fairweather et al., 2012; Gauntt and Huber, 2003). To date, no specific antiviral treatments are available for CVB3 infection, and clinical management remains primarily supportive, with limited efficacy in fundamentally inhibiting viral replication (Gao et al., 2024). Here, we show for the first time that AA suppresses CVB3 replication and systematically elucidate its molecular mechanism. *In vitro* experiments demonstrated that AA exerts dose-dependent inhibitory effects on CVB3 replication at non-cytotoxic concentrations, with an SI of 26.90, indicating a favorable safety profile and therapeutic window. Time-of-addition assays also demonstrated that AA mainly affects viral entry and intracellular replication, but does not have any significant effect on viral adsorption or direct virucidal activity. These results suggest that AA acts on host factors rather than viral components to exert its antiviral activity.

One of the most important findings of this study is the identification of RAN as a host factor required for CVB3 replication, and the demonstration that AA inhibits viral replication by downregulating RAN expression. The small GTPase family member, RAN, is traditionally associated with the mediation of nuclear-cytoplasmic transport (Güttler and Görlich, 2011; Sachweh et al., 2025). Recent findings have been found to implicate RAN in the life cycle of various viruses. As an example, HIV-1 Rev protein uses RAN-mediated nuclear export to promote viral mRNA transport (Behrens et al., 2017; Smith et al., 2025), while the influenza A virus NS2 protein controls nuclear export of viral ribonucleoprotein complexes by interacting with RAN (Cao et al., 2024). However, the role of RAN in CVB3 infection has not been much investigated. Using complementary loss- and gain-of-function approaches, we showed that CVB3 infection strongly upregulates RAN expression and that RAN levels positively correlate with viral replication efficiency. These findings establish RAN as a host dependency factor necessary for efficient CVB3 replication.

However, the exact molecular pathway of RAN upregulation during CVB3 infection should be explored. The observation that CVB3 induces multiplicity-dependent RAN expression raises the question of which signaling pathways are involved. Since various viruses hijack host signaling cascades such as mitogen-activated protein kinases (MAPK), nuclear factor kappaB (NF-kappaB), and phosphatidylinositol 3-kinase (PI3K)/protein kinase B (PKB/AKT) to regulate host genes expression (Chander et al., 2021; Hiscott et al., 2006; Kapoor et al., 2026; Moens, 2022), systematic screening with pathway-specific inhibitors could determine the major signaling nodes that cause virus-induced RAN upregulation. Furthermore, it remains to be determined whether RAN induction represents a general host response to *Enterovirus* infection or a strain-specific effect of CVB3 that may not apply to other CVB3 strains or other *Enterovirus* species.

Molecular docking, coupled with dynamics simulation research, uncovered the positive affinity between AA and RAN, and the protein-ligand complex was stable during 100 ns simulations. Interaction analysis identified hydrophobic contacts between AA and residues Phe35, Tyr39, and Ile126 of RAN, which may form a functional binding pocket. Although these computational results provide structural insight into the AA–RAN interaction, cellular-level functional validation is still needed.

This study validates that RAN is essential for restoring the nuclear localization of p-STAT1/2. Nevertheless, the classical STAT nuclear-cytoplasmic shuttling requires not only energy from the RAN system but also specific nuclear transport receptors such as chromosome region maintenance 1 (CRM1) to mediate nuclear export (Banninger and Reich, 2004; McBride and Reich, 2003). Even though we have seen that AA can facilitate the nuclear accumulation of STAT, it cannot be excluded that the virus could synergistically interfere with the nuclear-cytoplasmic balance of STAT through its effect on CRM1 functionality. Therefore, additional research is required to determine whether the effect of AA is entirely independent of the CRM1 pathway or indirectly corrects abnormal nuclear export by restoring RAN-GTP homeostasis, thereby providing a more comprehensive understanding of its regulatory mechanism in innate immunity.

Mechanistically, we found that AA regulates STAT signaling via RAN-dependent regulation. The core transcription factor complex of the IFN signaling pathway consists of STAT1 and STAT2; once activated, phosphorylated STAT1/2 heterodimerize, bind to interferon regulatory factor 9 (IRF9) to form the ISGF3 complex, and translocate to the nucleus to induce ISG transcription (Blaszczyk et al., 2016; Platanitis et al., 2019). Viruses have developed various mechanisms to hijack this pathway by interfering with STAT nuclear transport. For instance, paramyxovirus V protein directly interacts with STAT proteins and inhibits their phosphorylation (Horvath, 2004), whereas dengue virus non-structural protein 5 induces degradation of STAT2 (Ashour et al., 2009). Our observation that CVB3 infection causes cytoplasmic retention of p-STAT1/2 with a concomitant loss of nuclear p-STAT1/2 suggests that CVB3 employs a similar mechanism to evade the immune response. Interestingly, AA treatment rescued p-STAT1/2 nuclear localization in a RAN-dependent manner. Co-immunoprecipitation experiments showed physical interaction between RAN and p-STAT1/2, which provided biochemical evidence for RAN involvement in STAT nuclear transport. These findings align with a model in which RAN promotes p-STAT1/2 nuclear translocation under homeostatic conditions; CVB3 infection disrupts this process by upregulating or functionally altering RAN, thereby inhibiting antiviral signaling; and AA restores p-STAT1/2 nuclear trafficking by downregulating RAN expression or competitively binding to RAN, which reactivates ISG transcription and establishes an antiviral state.

Beyond restoring nuclear import, our data also reveal that AA treatment increases the total cellular levels of p-STAT1 and p-STAT2, as both cytoplasmic and nuclear fractions show higher signals compared to the virus-alone control. This observation suggests that AA may also enhance the phosphorylation of STAT1/2, an event that occurs upstream of nuclear translocation. The increased phosphorylation could result from several non-mutually exclusive mechanisms. First, AA-mediated downregulation of RAN might indirectly relieve viral suppression of the Janus kinase (JAK)-STAT pathway. If CVB3 infection suppresses STAT1/2 phosphorylation, reducing viral replication via AA would secondarily restore phosphorylation. Second, AA could directly modulate host signaling pathways that converge on STAT activation, such as the MAPK or PI3K/Akt cascades, which are known to cross-talk with JAK-STAT signaling. Third, because RAN has been implicated in the trafficking of signaling intermediates, it is conceivable that RAN dysregulation indirectly affects the kinetics or compartmentalization of the phosphorylation machinery. Further experiments using JAK inhibitors or direct measurement of JAK activity in the presence of AA would help distinguish between these possibilities.

The *in vivo* antiviral effect of AA was also confirmed by a viral myocarditis model of CVB3-induced mouse. AA treatment significantly improved survival rates, attenuated weight loss, and reduced myocardial viral loads. At the same time, AA inhibited the production of pro-inflammatory cytokines (TNF-α, IL-6, IL-1β) in heart tissue, reduced serum levels of cardiac injury markers (CK-MB and LDH), and ameliorated histopathological damage. These results suggest that AA confers cardioprotection through a dual mechanism: directly as an antiviral agent, and indirectly as an anti-inflammatory agent that suppresses excessive host immune responses. This dual action is particularly advantageous, as viral infection can cause tissue damage via both direct cytopathic effects and uncontrolled immune reactions.

Several limitations of this study should be acknowledged. First, the antiviral range of AA to other enteroviruses has yet to be defined. Since group B coxsackieviruses have six serotypes, which can all lead to human disease, evidence of broad-spectrum activity would significantly increase the translational value of AA. Second, the *in vivo* experiments used a prophylactic dosing regimen initiated soon after infection; future studies using therapeutic dosing regimens started at later time points would be more reflective of clinical situations, in which patients seek treatment after symptoms appear. Third, the present study only assessed short-term outcomes; long-term follow-up studies should be conducted to determine whether AA confers long-term benefits on cardiac function, fibrosis prevention, and heart failure prevention. Fourth, pharmacokinetic characteristics and long-term safety profile of AA need systematic assessment such as oral bioavailability, tissue distribution, and possible multi-organ toxicity with prolonged use.

Despite these limitations, the present study provides important insights of both scientific and translational significance. AA is an example of a host-directed antiviral strategy that targets the host factor RAN. It offers theoretical advantages over direct-acting antivirals, including a higher barrier to drug resistance and potential for broad-spectrum activity. As a natural product with a defined structure, abundant availability, and a favorable safety profile, AA represents a promising lead compound for further development.

## Conclusion

**FIGURE 6:**
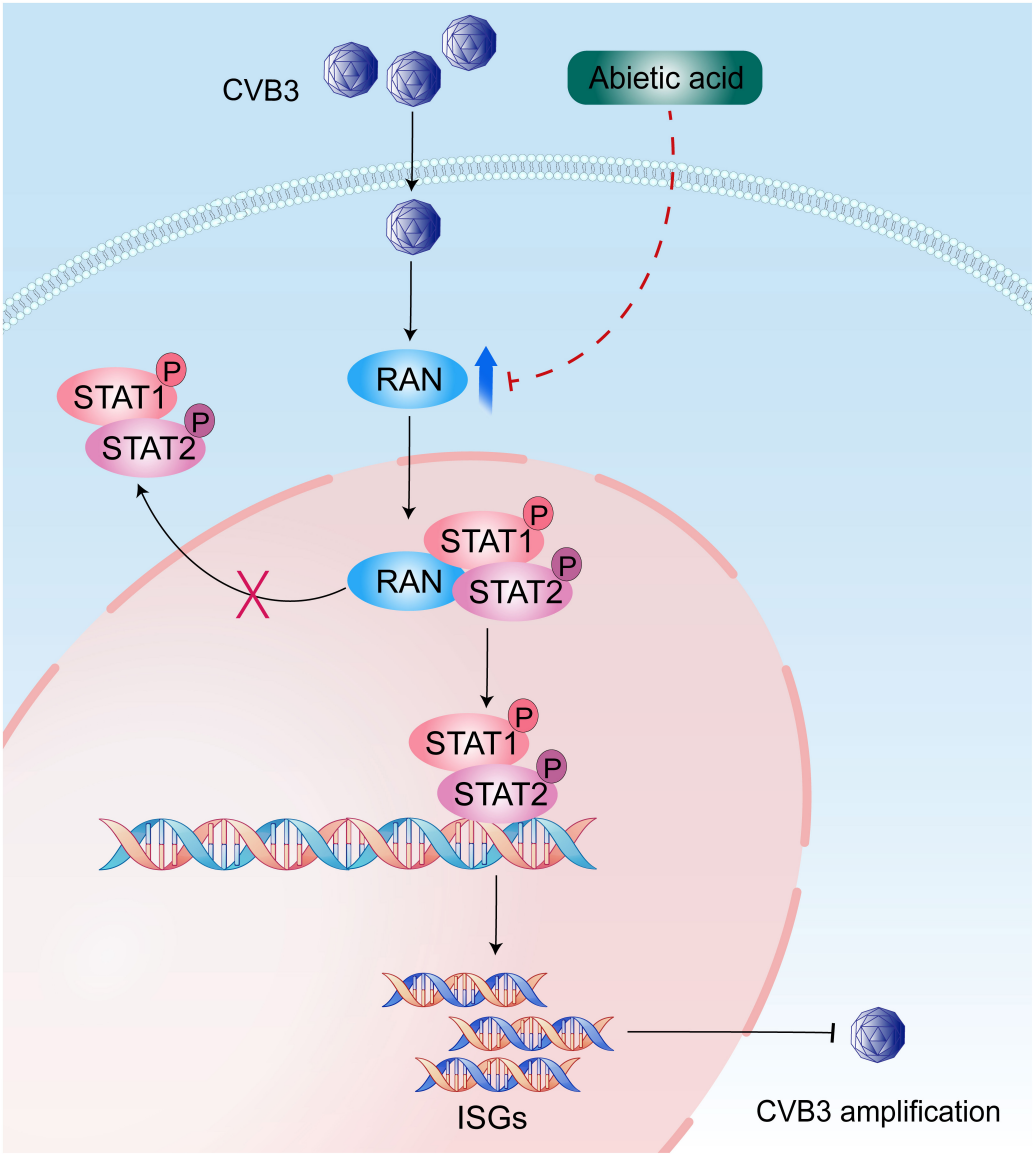
Schematic diagram of the anti-CVB3 mechanism of AA.

In summary, this study demonstrates that AA targets the host factor RAN, restores p-STAT1/2 nuclear translocation, enhances antiviral signaling, and effectively inhibits CVB3 replication both *in vitro* and *in vivo* (Figure 6). These results reveal a novel mechanism of AA-mediated antiviral activity and support its potential as a therapeutic agent against VMC.

## Data Availability

The raw data supporting the conclusions of this article will be made available by the authors, without undue reservation.
