# Supplementary material for: Abietic acid targets RAN to restrict coxsackievirus B3 infection
Source: Front Microbiol. 2026 Jun 16;17:1874898. doi: 10.3389/fmicb.2026.1874898 (PMC13315223; doi:10.3389/fmicb.2026.1874898)
Supplement: Supplementary file 1 [file Table_1.docx]

Table S1 Gene primers used in RT-qPCR

| Gene | Forward Primer | Reverse Primer |
| --- | --- | --- |
| CVB3 | GCACACACCCTCAAACCAGA | ATGAAACACGGACACCCAAAG |
| IL-1β (Mouse) | AAAGACCTCTATGCCAACACAGT | CTGACTTGGCAGAGGACAAAG |
| IL-6 (Mouse) | TAGTCCTTCCTACCCCAATTTCC | TTGGTCCTTAGCCACTACTTC |
| TNF-α (Mouse) | TCAAGTGGCATAGATGTGGAAGAA | TGGCTCTGCAGGATTTTCATG |
| GAPDH (Mouse) | AGGGCATCTTGGGCTACAC | CATACCAGGAAATGAGCTTGA |
| GAPDH (Human) | GCACCGTCAAGGCTGAGAAC | TGGTGAAGACGCCAGTGGA |
| BST2 (Human) | CACACTGTGATGGCCCTAATG | GTCCGCGATTCTCACGCTT |
| OAS3 (Human) | GCTTCAAGAGCTATGTGGACC | GGAAACGTGAGTCTCAGACCA |
| ISG20 (Human) | CGCAGATCACCCAGAAGATCG | TTCGTCGCATTTGTCCACCA |
| IFIT1 (Human) | AGAAGCAGGCAATCACAGAAAA | CTGAAACCGACCATAGTGGAAAT |
| IFIT2 (Human) | AAGCACCTCAAAGGGCAAAAC | AAGCACCTCAAAGGGCAAAAC |
| CXCL10 (Human) | CCTGCTGGGTCTGAGTGGGA | GATAGGCT CGCAGGGATGAT |
| RAN (Human) | GAAAGTGAAGGCGAAATCCATTGTC | CAACCTCTAGTCGTGCTCATACTG |
